# Cognitive processing and body balance in elderly subjects with vestibular dysfunction

**DOI:** 10.1590/S1808-86942012000200014

**Published:** 2015-10-20

**Authors:** Giovanna Cristina dos Santos Caixeta, Flávia Doná, Juliana Maria Gazzola

**Affiliations:** a Physical therapist. Master's degree in rehabilitation of body balance and social inclusion, São Paulo Bandeirante University (Universidade Bandeirante de São Paulo or UNIBAN-Brazil). (Physical therapist); b Physical therapist. Post-doctoral student in the Movement Disorders Sector, graduate program in neurology and neuroscience, Sao Paulo Federal University (Universidade Federal de São Paulo or UNIFESP). (Faculty member in the master's degree program for rehabilitation of body balance and social inclusion, UNIBAN-Brazil); c Physical therapist. Master and doctoral degrees in science, Otorhinolaryngology Department, UNIFESP. (Faculty member in the master's degree program for rehabilitation of body balance and social inclusion, UNIBAN-Brazil)

**Keywords:** aged, cognition, dizziness, postural balance, vestibular diseases

## Abstract

Abnormal body balance and cognitive dysfunction may develop in elderly patients with chronic vestibular dysfunction.

**Aim:**

To evaluate the relationship between cognitive processing and body balance in elderly patients with chronic peripheral vestibular disease. Type of Study: Cross-sectional.

**Methods:**

Seventy-six patients (≥ 60 years) with chronic peripheral vestibular dysfunction and dizziness for more than three months were enrolled. The tests for investigating body balance were: the Berg Balance Scale (BBS), Dynamic Gait Index (DGI), Timed Up and Go Test (TUGT) Timed Up and Go Test modified (TUGTm); the Mini Mental State Examination (MMSE), Test Clock (RT,) and Verbal Fluency Test (VF) were applied for assessing cognition.

**Results:**

The mean age was 69.03 years (SD=6.21 years); most were female (82.9%). There was a significant negative correlation between the MMSE and the TUGT (ρ=-0.312; *p*=0.01), MMSE and TUGTm (ρ=-0.306; *p*=0.01), FV and TUGT (ρ=-0.346; *p*=0.01), and FV and TUGTm (ρ=-0.536; *p*=0.01); there was a significant positive correlation between the TR and BBS (ρ=0.343; *p*=0.01), TR and DGI (ρ=0.298; *p*=0.01), FV and BBS (ρ=0.299; *p*=0.01), and FV and DGI (ρ=0.306; *p*=0.01).

**Conclusion:**

Elderly patients with chronic peripheral vestibular disease and worse performance in body balance tests have functional impairment in cognitive skills.

## INTRODUCTION

Ageing entails several physical and functional changes including an altered body balance commonly accompanied by a decline in physical and cognitive abilities^1^. Cognition here may be defined as the ability to use a variety of learned abilities to respond adaptively to external demands^2^.

Cognitive decline by ageing – characterized by loss of concentration and short-term memory loss – is often seen by clinicians in patients with vestibular diseases; it is more evident in tasks that require speed and inductive reasoning^3^, ^4^, ^5^, ^6^. Thus, loss of memory and difficulty to concentrate may accompany vestibular diseases and such patients may present associated body unbalance^3^, ^4^, ^5^. All components of postural control (sensory, effector, central processing, and cognition) are affected by ageing. It is therefore a complex task to separate the effects of senescence from the subtle symptoms of subclinical diseases and the changes in lifestyle that accompany ageing^7^, ^8^, ^9^. The body system's compensatory mechanism is compromised because of accumulated losses that may affect postural control, which increases instability and the risk of falls^7^, ^8^, ^9^, ^10^.

When proprioceptive and visual information is absent or inaccurate, the central nervous system (CNS) recognizes the vestibular system as the main source of sensory information. Elderly patients with vestibular diseases, however, are unable to adequately apply such information, and may present dizziness, unbalance, increased body oscillation, and gait abnormalities. Several otoneurological symptoms arise proportionally as age increases; among these symptoms are hearing loss, tinnitus, and occasional falls. In these cases, dizziness and/or body unbalance are due to primary or secondary functional disorders of the vestibular system^11^, ^12^, ^13^.

Some authors have suggested that the hippocampus system manages and maintains the flow of information in the cognitive system for individuals to carry out specific objectives or functions. This system may be compromised in patients with vestibular disorders, in turn affecting the individual's attention system^14^, ^15^.

Even though the literature contains several papers on the relationship between cognitive processing disorders and abnormal body balance in elderly individuals with chronic peripheral vestibular disease, the nature of these relationships has yet to be elucidated^3^, ^4^, ^5^, ^6^, ^13^. The purpose of this study is to investigate the relationship between body balance and cognitive processing in elderly individuals with chronic peripheral vestibular disease.

This study may be justified by complaints of poor attention, concentration, and memory in patients with peripheral vestibular disease and a paucity of information to support the relationship between attention deficits – maintained by the hippocampus system – and body balance in these patients. It may therefore be possible to provide support for recovery of function in these individuals.

## MATERIAL AND METHODS

A contemporary cohort cross-sectional study was made of elderly individuals with a diagnosis of chronic peripheral vestibular diseases that complained of chronic dizziness and/or body unbalance. The institutional review board of our university approved this study (protocol no. 236–8).

Male and female patients aged 60 years or more with a diagnosis of chronic peripheral vestibular disease and complaints of dizziness and/or functional body unbalance for at least three months^11^, ^12^, ^13^ with at least incomplete basic education were enrolled.

Individuals with advanced osteomuscular degenerative diseases using walkers and walking sticks and patients with severe visual or hearing loss were excluded from the study.

Patients were referred for assessment of cognition and functional body balance after going through medical and phonoaudiological evaluations to reach a diagnosis of chronic peripheral vestibular disease. The Mini Mental State Examination (MMSE)^16^, ^17^, the Clock Test (CT)^18^, ^19^, ^20^, ^21^, and the Verbal Fluency Test (VFT) were applied to evaluate cognitive processing^2^, ^22^, ^23^, ^24^. During a preliminary phase for subsequent tests, the MMSE^25^ was applied – it evaluates time and spatial orientation, word recording, attention, calculation, evocation, language, and visual/constructive praxis. It has been used with success to evaluate cognition in elderly individuals^16^, ^17^, ^18^, ^19^, ^20^.

The exclusion criteria when evaluating cognition was 17 points for subjects with up to 9 years of schooling, and 23 points or more for elderly subjects with over 9 years of schooling^16^, ^24^. Illiterate patients that did not score within the set cut point for schooling were also excluded from the study.

CT^18^, ^19^, ^20^ was also used because it evaluates several functions of cognition, such as verbal understanding, praxis, visual/spatial function, attention, memory, and executive functions. It consists of asking subjects to draw the numbers and hands of a clock at 11:10 h, without mentioning the need for hands. The score ranges from zero (absolute inability to represent a clock) to 5 (a perfect clock without errors).

Marking that particular time on the clock aims to draw the hands using two visual fields – the superior quadrants – or “temporal fields”. Fuzikawa^21^ has stated that this criterion as adopted because 11:10 h is the layout with the highest sensitivity to detect neuropsychological abnormalities.

The cutoff points for our study were those proposed by Shulman^20^. The cutoff score was 3.5 points for less educated elderly individuals; it was 4.5 points for more educated patients. Again, Fuzikawa^21^ states that scores below 4 points require additional investigation. This test is essential for diagnosing dementia. A command condition requires enough language to understand verbal instructions, semantic memory to remember the shape of a clock, and executive functions from the frontal lobe.

VFT^2^, ^22^, ^23^ is used to assess semantic memory for predefined knowledge, such as that about animals. Studies of elderly individuals have used the following criteria for this test: the cutoff score is 9 animals per minute for less educated subjects (less than 9 years of schooling); the cutoff score is 13 animals per minute for more educated subjects (more than 9 years of schooling^2^, ^22^.

The *Berg Balance Scale (*BBS) was applied to evaluate functional body balance. Berg et al.^26^, ^27^ validated this scale and Miyamoto et al.^28^ adapted it culturally to Brazil. The BBS consists of a scale with 14 common daily tasks that require static and dynamic body balance – reaching, turning, transferring oneself, remain standing, and standing up. It is possible to use the test to check a patient's ability, to monitor progression in exercises, and to assess the effectiveness of therapy^26^, ^27^, ^28^. Progressively more points are subtracted if subjects do not reach a set time or distance or if they require supervision for carrying out a task, seek external support, or receive help from an examiner. The lowest response score was recorded for each item.

Tasks were evaluated by observation; scores ranged from 0 to 4, totaling 56 points; a higher score in the BBS means a better functional body balance performance. The cutoff value in this study was 48 points, which was a predictor of falls in elderly patients with vestibular disease, according to Medeiros^29^. This author evaluated 76 elderly individuals with peripheral vestibular disease and a complaint of chronic dizziness; the 48-point cutoff level was the most clinically relevant for identifying elderly individuals with vestibular disease that reported falls.

The *Dynamic Gait Index (*DGI)^30^, ^31^, ^32^ test is used for measuring gait dysfunction in adults and elderly individuals, and to adjust steps together with functional tasks such as head movements, velocity changes, velocity changes around objects, and climbing up and down stairs. Qualitative and quantitative assessment of gait consists of eight tasks, as follows: gait over a flat surface, velocity changes during gait, gait with horizontal rotation of the head, gait with vertical rotation of the head, gait and rotation, passing over a hurdle, walking around an obstacle, and climbing up and down stairs.

Each patient was classified on an ordinal scale with four categories and scored according to the performance in each functional task; each was graded on an ordinal scale from 0 to 3, totaling 24 points. Higher test scores meant a better performance in the DGI. A total score of 19 or less in the DGI was associated with a higher risk of falls in elderly individuals^28^, ^30^, ^32^.

The *Timed Up and Go Test (*TUGT) was used to assess mobility, dynamic body balance, and the risk of falls in elderly subjects with vestibular diseases. This test yields information about gait and mobility by using components that comprise daily activities, such as standing up from a sitting position, sitting, walking, and changing the direction of gait^33^, ^34^, ^35^. The TUGT is widely used because it is easy to apply. Together with a chronometer (Casio®), within seconds it quantifies functional mobility for carrying out a given task; it measures how many seconds it takes for a person to stand from a sitting position on a standard chair with arm and back support, to walk three meters, to turn, to return to the chair, and to sit again. Elderly subjects that take more than 13.5 seconds to carry out the TUGT are at a higher risk for falls^34^.

The modified version of the TUGT contains a cognitive task to assess the influence of attention demand on the body balance of elderly individuals^8^, ^33^, ^34^, ^35^, ^36^. The test procedure is similar to the TUGT with the addition of a procedure in which names of animals are evoked after a verbal fluency test. It starts with the following instruction: “Tell me the names of all the animals you can remember.” When a signal is given, while seated, an elderly subject starts to evoke the names of animals and at the same time stands up, walks a 3-meter straight line, returns, sits down, and rests his or her back on the same chair. The patient is asked not to stop evoking the names of animals while carrying out the test and to do it as fast as possible. Elderly individuals that take more than 15 seconds to carry out the TUGTm are at a higher risk for falls^35^.

Data were gathered and analyzed only by the main researcher to assure confidentiality, and were fed into a database in the *SPSS 15.0 for Windows (Statistical Package for Social Sciences,* version 15.0) and the *BioEstat* 2007.

The decision about the sample size was made after analyzing similar studies and after a statistical calculation.

Because of the sample size, criteria in the following website were applied for a descriptive analysis of the data: http://www.lee.dante.br/pesquisa/amostragem/qua_1_media_est.html#err-max; the maximum estimated error around the mean is 12%. The sample size was 72 patients. The *Kolmogorov-Smirnov test was applied to calculate the normal distribution of data for the statistical correlation analysis. This test is used for samples over* 48 data. A significance level of *p*<0.05 indicates that the distribution of data differs significantly from a normal distribution.

Non-parametric test were applied because of data asymmetry and variability, and because data were not distributed normally. Spearman's test (ρ) was applied to check these associations; it is used for ordinal or higher-level variables when comparing quantitative scores^37^. Correlation analyses were made among the cognitive tests (MMSE, VFT, and CT) and the functional body balance tests (BBS, DGI, TUGT, and TUGTm).

## RESULTS

The mean age of patients was 69.03 years (SD=6.21 years). The maximum age was 86 years. Table 1 presents the social/demographic data.

**Table 1:** Social/demographic data of 76 elderly subjects with chronic peripheral vestibular disease

	Categories	Relative frequency (n)	Absolute frequency (%)
Gender	Female	63	82.9
	Male	13	18.8

Education level	IBE	55	72.4
	Secondary education	14	18.4
	Higher education	07	9.2

Age group	60–64 years	20	26.3
	65–69 years	24	31.6
	70–74 years	21	27.6
	75–80 years	07	9.2
	81 years or more	04	5.3

IBE: incomplete basic education.

### Assessment of Cognition

Table 2 shows the performance of patients in the cognitive tests as descriptive data. Table 3 indicates the MMSE, VFT, and CT scores according to the normalcy range and education level. In the MMSE, the cognition of 47 patients (64.5%) was preserved. In the VFT test, 12 less educated patients (15.8%) named 0 to 8 animals/minute), and 16 more educated patients (21.1%) named up to 13 animals/minute. This shows that elderly patients with more education had results that suggested cognitive abnormalities in the verbal fluency test.

**Table 2:** Mean values, standard deviation, minimum and maximum, of cognitive tests: MMSE, C T, and VFT in 76 elderly subjects with chronic peripheral vestibular disease.

Variables	Mean	SD	Minimum	Maximum
Mini Mental State Examination	23.88	2.67	17	29
Verbal Fluency Test	11.44	3.19	5	19
Clock Test	3.72	1.36	1	5

**Table 3:** Absolute and relative frequencies in the classification of scores in the MMSE, the VFT, and the CT in 76 elderly subjects with chronic peripheral vestibular disease.

	Categories	Absolute frequency (n)	Relative frequency (%)
Mini Mental State Examination	0–23 points: poor cognition	27	35.5
	24–30 points: intact cognition	49	64.5

Verbal Fluency Test	Less educated: up to 8 names of animals/minute (abnormal)	12	15.7
	Less educated: 9 names of animals/minute or more (normal)	43	56.6
	More educated: up to 13 names of animals/minute (abnormal)	16	21.1
	More educated: 14 names of animals/minute or more (normal)	05	6.6

Clock Test	More educated: Over 4.5 points	13	17.1
	More educated: Below de 4.5 points	11	14.5
	Less educated: Over 3.5 points	28	36.8
	Less educated: Below 3.5 points	24	31.6

In the CT, 28 less educated patients (36.8%) were within normal limits, and 24 patients (31.6%) had results that suggested cognitive decline. Among more educated patients, 13 (17.1%) had scores within normal limits, and 11 (14.5%) had signs of compromised cognition.

### Assessment of Functional Body Balance

The mean score in the functional body balance assessment was: BBS – 51.13 (SD=6.03), ranging from 17 to 56 points; 59 patients (77.6%) scored from 49 to 56 points – a group at no risk for falls; 17 patients (22.4%) scored from 0 to 48 points – a group at risk for falls (Table 4).

**Table 4:** Absolute and relative frequencies of the variables BBS, DGI, TUGT, and TUGTm in 76 elderly subjects with chronic peripheral vestibular disease.

	Categories	Absolute frequency (n)	Relative frequency (%)
BBS	0 to 48 points	17	22.4
	49 to 56 points	59	77.6
DGI	0 to 19 points	22	28.9
	20 to 24 points	53	71.1
TUGT	up to 13.5 seconds	72	94.7
	13.5 or more seconds	4	5.3
TUGTm	up to 15.0 seconds	62	81.6
	15.0 or more seconds	14	18.4

BBS: Berg Balance Scale

DGI: Dizziness Gait Index

TUGT: Timed Up Go Test TUGTm: Timed Up Go Test Modified

The mean score in the DGI was 20.91 (SD=2.71), ranging from 14 to 24 points; 22 patients (28.9%) scored less than 19 – a group at risk for falls (Table 4); 53 patients (71.1%) belong to a group at a lower risk for falls, scoring 20 to 24 points.

In the TUGT, the mean time for carrying out the test was 9.38 seconds (SD=2.07), ranging from 6.05 to 15.20 seconds.

In the TUGTm, the mean time for carrying out the test was 12.08 seconds (SD=3.53), ranging from 6.11 to 22.78 seconds.

In the TUGT, most patients (72 or 94.7%) carried out the test within 13.5 seconds. In the TUGTm, 62 patients (81.6%) completed the test within 15.0 seconds, as shown in Table 4.

### Correlation Analysis of Cognition × Body Balance

A low but significant negative correlation was found between the MMSE and the TUGT (ρ=-0.312; *p*=0.01) and between the MMSE and the TUGTm (ρ=-0.306; *p*=0.01). Thus, a higher MMSE score correlates with less time required for carrying out the TUGT and TUGTm tests. There was no statistically significant correlation among the MMSE, the BBS, and the DGI (Table 5). A low but statistically significant positive correlation was found among the CT, the BBS (ρ=0.343; *p*=0.01) and the DGI (0.298; *p*=0.01). There was no significant correlation among the CT, the TUGT, and the TUGTm (Table 5). The data revealed a low but significant positive correlation among the VFT, the BBS (ρ=0.299; *p*=0.01), and the DGI (ρ=0.306; *p*=0.01). There was a low but significant negative correlation between the VFT and the TUGT (ρ=0.346; *p*=0,01), and a moderate significant negative correlation between the VFT and the TUGTm (ρ=-0.536; *p*=0.01) (Table 5).

**Table 5:** Correlation among cognitive tests (MMSE, CT, and VFT) and functional body balance tests (BBS, DGI, TUGT, and TUGTm) in 76 elderly subjects with chronic peripheral vestibular disease.

Categories	Spearman's correlation coefficient (ρ)
MMSE X BBS	0.224
MMSE X DGI	0.175
MMSE X TUGT	−0.312**
MMSE X TUGTm	−0.306**
CT X BBS	0.343**
CT X DGI	0.298**
CT X TUGT	−0.225
CT X TUGTm	−0.200
VFT X BBS	0.299**
VFT X DGI	0.306**
VFT X TUGT	−0.346**
VFT X TUGTm	−0.536**

MMSE: Mini Mental State Examination.

CT: Clock Test.

VFT: Verbal Fluency Test.

BBS: Berg Balance Scale.

DGI: Dynamic Gait Index.

TUGT: Timed Up and Go Test.

TUGTm: Timed Up and Go Test modified.

** Significant correlation (*p*=0.01).

## DISCUSSION

Patients with vestibular disorders commonly report cognitive deficits, such as poor attention, concentration, memory, and spatial orientation. These losses become evident in tasks that require speed, attention, and inductive reasoning^3^. Other authors, however, have questioned this published material, which makes it difficult to develop adequate inferences on the existence of associations between vestibular diseases and cognition^4^, ^38^, ^39^, ^40^.

The mean age in our sample was 69.03 years; most subjects were in the 65–69 year age range (31.6%). The sample consisted mostly of female subjects (82.9%). These findings are similar to those in other published studies – Gushiken et al. (67.64%)^38^, Peixoto (77.5%),^36^ and Gazzola et al. (68.3%)^12^.

The screening of cognitive function showed that the mean score in the MMSE was 23.88 points; 47.0% of patients (64.5%) scored from 24 to 30 points, and 27 patients (35.5%) had cognitive decline. Bertolucci et al.^25^ have suggested that scores in the 24 to 30 point range are associated with no cognitive deficit. Peixoto^36^ and Gazzola et al.^12^ found similar values in their samples – respectively 24.10 and 24.45 points.

The hippocampus system has to be intact for patients to orient themselves in space and to preserve functional abilities; central commands from the hippocampus need to be allocated. The MMSE is a screening test for assessing evocation memory, temporal spatial orientation, and visual and spatial abilities. Higher scores in the MMSE test indicate a better functional performance.

The data showed that there was a negative correlation between the MMSE and functional balance tests such as the TUGT and the TUGTm. Intact cognitive function is associated with better mobility and control of posture for carrying out the tasks in the TUGT and the TUGTm (a double tasks – mobility and verbal fluency). This is possible because the MMSE investigates executive and language functions.

The TUGT provides information about mobility because it uses components of daily life – standing from a seated position, walking, changing the direction of gait – and a cognitive task to evaluate the effect of attention demand on balance in elderly subjects.

In the TUGT it is possible to show that most patients carried out the test within 13.5 seconds; most patients performed the TUGTm within 15.0 seconds. Nordin et al.^34^ consider the TUGT an important test for predicting falls in elderly subjects, and that lower MMSE scores correlate with a higher rate of falls.

These findings show that most of the study sample (81.6%) had no loss of functional body balance when it was associated with cognitive tasks, and therefore had no risk for falls.

The CT aims to assess temporal and frontal lobe functions and semantic memory in elderly subjects. The CT is an excellent tool for checking the ability to plan, and temporal and spatial orientation, abilities that are important for body balance. The test can yield more complex assessments relative to the MMSE.

The CT in this study was 3.72 (SD=1.36), indicating that patients in the sample had no loss in understanding, memory, spatial notions, abstraction, planning, concentration, and concentration and visual/constructive abilities. Shulman et al.^19^ and Shulman^20^ have suggested that there are more cognitive changes in patients with less education.

Our data showed a significant negative correlation between the CT and the DGI – higher points in the neuropsychologic test correlated with a better performance in gait and lower risk for falls. Patients with vestibular dysfunction may find it difficult to allocate cognitive processing during gait and while carrying out tasks that require head movements^28^, ^31^, ^41^.

As noted above, the DGI assesses and quantifies the ability of subjects to integrate and adjust steps while carrying out functional tasks. Our data showed that the mean score in this test was 20.91 (SD=2.91), and that 71.1% of patients were not at a higher risk for falls when scoring 20 to 24 points. Gazzola^12^ and Whitney et al.^31^ have shown mean scores of 19.35 (SD=3.82) and 16.7 (SD=5.1) each.

Whitney et al.^32^ have stated that demand for attention during gait may make it more difficult for patients in the sample to carry out the DGI, especially tasks that require movements of the head. Such movements generate symptoms of bodily instability and dizziness, reduce visual and bodily stability, and increase the risk for falls in the study population.

The VFT – word groups spoken during one minute, such as the names of animals – requires verbal memory, and is related with the temporal lobe. On the other hand, exchanges require mental and cognitive flexibility, and are related with the frontal lobe to keep varied cognitive information active for a specific end^2^, ^22^, ^23^. A regulatory and attention system is needed to control the flow of cognitive information so that an intended action is carried out.

In the VFT a profile of the semantic memory of patients with vestibular disease may be drawn; the mean number of animals evoked within a minute was 11.44, which was similar to Peixoto's^36^ results (11.37 animals per minute).

We found a negative correlation among the VFT, the TUGT and TUGTm, which suggests that when elderly patients with vestibular diseases carry out double tasks, attention and cognitive processing are compromised while a body balance test is done – an indication that both systems interfere with each other^3^, ^36^. These findings suggest that vestibular disorders may cause difficulties for cognitive processing^39^, ^40^, ^41^. A possible explanation for this is a likely deficiency in spatial processing due to vestibular injury. According to this argument, vestibular injury may cause atrophy in the hippocampus system, which in turn projects this deficiency to cognitive processing because of its particular effect on memory.

The VFT also revealed a significant association with body balance tests – the BBS and the DGI. It became clear that a superior performance in body balance tests correlated with a better score in the VFT.

The assessment of functional body balance by the BBS showed that the mean values was 51.13 points (SD=6.03), corroborating the findings of Gazzola et al.^12^ – their mean BBS value was 50.21 points (SD=6.01). These results, however, differed in Whitney et al.'s^32^ study; in this case, these authors found a mean BBS value of 46.5 points (SD=7.0) in patients aged over 60 years that presented vestibular disease. In our study, 59 patients (77.6%) scored from 47 to 54 points – a lower risk for falls, and 17 patients (22.4%) scored 0 to 46 points – a higher risk for falls.

We may infer that the VFT and CT, among cognitive tests, may be important for indicating abnormal functional balance in elderly patients with vestibular diseases; thus, an assessment of cognition may be required – a point usually not approached in conventional evaluations.

Cognitive tests make it possible to assess spatial orientation, attention, and language in greater detail; if applied together with static and dynamic functional tests, they may facilitate clinical decision-making. The study showed that the MMSE did not correlate with functional body balance tests, which may have occurred because it is an initial screening test that should not be used as the sole investigation tool.

Additionally, attention and spatial orientation within a preset distance in the TUGT indicate situations that are assessed in the MMSE language, verbal commands, and attention. The VFT and the CT contain elements that deepen the assessment of other aspects of cognition.

In parallel to these findings, our study revealed a correlation among the VFT, the TUGT, and the TUGTm, suggesting the VFT may be an investigation tool in dynamic and in language tests.

It should be noted that healthy elderly patients with no altered body balance were not evaluated to assess and quantify cognitive processing in elderly subjects with no peripheral vestibular disease; this is a limitation of this study.

We were able to show that when cognitive processing – specifically semantic memory – is intact, patients with peripheral vestibular disease are better able to control body balance. This is probably because these patients are more able to plan and execute motor responses and thereby control body balance.

We cannot state, based on our study, that planning of motor activity and execution of motor responses are the only causes of a superior performance in patients with intact cognition. There are no data from electronystagmography and the etiologic diagnosis to support this hypothesis. It is, however, likely that vestibular injury in patients with a worse performance is more extensive compared to that in patients with a good performance. Thus, lack of such information could be the main cause of disordered body balance.

## CONCLUSION

We were able to show that there is a relationship between cognitive processing and body balance disorders in patients with chronic peripheral vestibular disease. The associations were: between the CT and the VFT, in the body balance evaluations, based on the total scores of the BBS and DGI. It was also clear that there were significant negative correlations between the MMSE and VFT, and the TUGT and TUGTm.
